# Skeletal, dentoalveolar and soft tissue changes after stabilization splint treatment for patients with temporomandibular joint disorders

**DOI:** 10.1186/s12903-024-04260-3

**Published:** 2024-04-20

**Authors:** Saba Ahmed Al-hadad, Madiha Mohammed Saleh Ahmed, Yunshan Zhao, Lu Wang, Wanqing Hu, Chushen Li, Xi Chen, Maged Sultan Alhammadi

**Affiliations:** 1 Department of Stomatology, The First Affiliated Hospital of Xi’an Jiaotong University, Xi’an, Shaanxi 710061 People’s Republic of China; 2 Department of Orthodontics and Dentofacial Orthopedics, Faculty of Dentistry, Ibb University, IBB, Republic of Yemen; 3 Department of Orthodontics and Dentofacial Orthopedics, Faculty of Dentistry, Aden University, Aden, Republic of Yemen; 4 Orthodontics and Dentofacial Orthopedics, Department of Preventive Dental Sciences, College of Dentistry, Jazan University, Jazan, Saudi Arabia

**Keywords:** Stabilization splint, Temporomandibular joint disorders, Dentoalveolar, Skeletal, Cone-beam computed tomography

## Abstract

**Background:**

Temporomandibular disorder (TMD) is a grouping of heterogeneous disorders with multifactorial origins. Stabilization splints (SS) have demonstrated an acceptable treatment effect in TMD. The possible changes at the skeletal, dental, and soft tissue levels need to be addressed to evaluate the benefit/risk ratio of this therapeutic procedure. Accordingly, this study aimed to three‑dimensionally evaluate skeletal, dentoalveolar and soft tissue changes after SS treatment for patients with TMD.

**Methods:**

This retrospective study included 74 adult patients with myofascial and/or intra-articular disorders (25 males and 49 females), with an average age of 22.88 ± 4.8 years, who underwent SS treatment. Pre- and post-treatment Cone beam computed tomography were analysed using Invivo 6.0.3 software. The primary outcome was the vertical skeletal and dentoalveolar changes, while the secondary outcomes were the anteroposterior skeletal, dentoalveolar and soft tissue changes. Paired t-test and Wilcoxon rank sum test were used for statistical analyses.

**Results:**

For the primary outcome; skeletally, there was a significant increase in mandibular plane inclination (difference: 0.82°±1.37), decrease facial height ratio (difference: 0.45%±1.07) and at the dentoalveolar level, the inclination of the functional (FOP-SN, FOP-FH) and bisecting (BOP-SN, BOP-FH) occlusal planes exhibited a significant increase too (difference: 0.38 ± 1.43°, 0.49 ± 1.62°, 0.44 ± 1.29° and 0.41 ± 1.17°, respectively) and also a decrease in the overbite (difference: -0.54 ± 0.83). For the secondary outcomes; there was a significant decrease in mandibular position (SNB) (difference: 1.60 ± 1.36°) and increase in the overjet (difference: 0.93 ± 1.04, *p* < 0.001) and a significant lower lip retrusion (difference: 0.33 ± 1.01 mm *p* < 0.01), was observed too.

**Conclusions:**

SS therapy resulted in significant vertical skeletal and dentoalveolar changes that were manifested mainly by facial height ratio, mandibular and occlusal plane changes, and to a lesser extent, significant anteroposterior skeletal, dentoalveolar, and soft tissue changes in the form of mandibular position, increased overjet and a more retrusive lower lip. These changes should be considered during patients’ selection prior to initiating SS therapy.

## Introduction

The term temporomandibular disorders (TMD) is broad and refers to various musculoskeletal and neuromuscular disorders that affect the temporomandibular joint, the masticatory muscles and associated tissues [1, 2]. TMD is the second most common musculoskeletal disorder causing pain and disability [3, 4]. Treatment options for TMD can vary depending on the severity and cause of the problem. These treatment options include invasive interventions such as surgical procedures like arthroscopy and open TMJ surgery [5] or minimally invasive approaches such as injections with hyaluronic acid, corticosteroids or platelet-rich plasma, which aim to reduce pain and inflammation [6, 7]. Alternatively, there are non-invasive treatments such as manual therapy, psychotherapy, physiotherapy, short-term pharmacotherapy and occlusal splints [8–11]. Occlusal splints are commonly used of a non-invasive treatment for patients with TMD [12], and among occlusal splint types, Stabilization splints are one of the most effective [13]. Stabilization splints play a crucial role in the treatment of various oral and extraoral conditions such as temporomandibular joint disorders (TMD), myofascial pain, bruxism, headaches and migraines [11, 14–16]. They are also known as muscle relaxants as they can effectively relieve muscle pain [1, 17] and allow the condyles to rest in their most musculoskeletally stable posture by reproducing functional occlusion [18].

Skeletal and dentoalveolar changes can be influenced by various factors, including age, genetics, and environmental factors [19, 20]. Studies have found that dental appliance therapies can also result in dentoalveolar and skeletal changes [21, 22]. However, some of these studies [23, 24] showed that the appliances lead to dentoalveolar changes, with little or no skeletal influence. In a study conducted by Oh et al. [25], dentoskeletal factors were examined in patients with TMD to predict the development of anterior open bite after SS treatment, and they found that the use of SS can induce the occurrence of anterior open bite after treatment.

Previous research revealed a functional relationship between masticatory muscle closure and occlusal plane (OP) inclination, which is a crucial occlusal indicator and is one of the elements influencing masticatory movement [26]. Changing the OP inclination can affect mandibular positioning and the adaptive response of the condyles; this response plays an essential role in establishing various dentoskeletal frames [27]. A previous study showed that the OP inclination in the sagittal plane reflects an acquired harmonic relationship between the craniomandibular configuration and function [28].

Magdaleno and Ginestal [29] mentioned in their study, which included three cases, several possible factors that could explain the occlusal changes that occur after the application of splints. These occlusal changes include mandibular position, masticatory muscle activity and occlusal load distribution in the vertical dimension. Other case report studies have noted potential changes following SS treatment, such as mandibular rotation and bite adjustments, which have implications for diagnosis and orthodontic planning [30, 31]. However, it is essential to know that most of these studies were primarily case reports and did not comprehensively examine all skeletal and dentoalveolar changes following SS treatment. So, to address that, this study aims to perform a thorough three-dimensional quantitative analysis of these changes in a group of TMD patients, taking into account a wider range of comprehensive variables and larger sample size to provide valuable insights to guide clinical diagnosis and treatment planning.

The possible changes at the skeletal, dental, and soft tissue levels need to be analyzed in both the vertical and anteroposterior directions to evaluate this therapeutic procedure’s risk-benefit ratio. The purpose of the present study was to primarily examine vertical skeletal and dentoalveolar changes and, secondarily, anteroposterior skeletal, dentoalveolar, and soft tissue changes after SS therapy.

## Materials and methods

### Study design

This retrospective clinical study was designed and conducted at the First Affiliated Hospital of Xi’an Jiaotong University in Xi’an, Shaanxi Province, China. It was conducted in accordance with the principles of the Helsinki Declaration. This study obtained ethical approval (No. XJTU1AF2022LSK-027) from the hospital’s Institutional Review Board prior to data collection and analysis. All of the participants provided written informed consent before participating in the study.

### Sample

#### Inclusion criteria

(1) age ranging within 18–33 years; (2) dentition meeting the retention requirements of SS and healthy periodontal condition; (3) all permanent teeth, except for the third molar, are erupted; (4) subjects presenting with myofascial and/or intra-articular disorders diagnosis based on clinical data collected using the Diagnostic Criteria for TMD (DC/TMD) Axis I; in which TMD could be divided in Group I: muscle disorders (including myofascial pain with and without mouth opening limitation); Group II: including disc displacement with or without reduction and mouth opening limitation; Group III: arthralgia, arthritis, and arthrosis [32]; (5) a discrepancy that is larger than 1 mm between centric relation (CR) and centric occlusion on the vertical plane and a discrepancy of more than 0.5 mm on the transverse plane [33]; (6) adherence with the treatment requirements and willingness to cooperate with the treatment plan; (7) completed SS treatment and existence of high-quality cone-beam computed tomography (CBCT) scans before and after treatment and (8) normal development of craniomaxillofacial structures.

#### Exclusion criteria

(1) history of trauma to the craniomaxillofacial region; (2) idiopathic condylar resorption in the active phase; (3) history of orthognathic treatment, orthodontic treatment or surgery of the temporomandibular joint; (4) abnormal psychological or mental behaviour and (5) presence of systemic immune system diseases or metabolic diseases.

#### Intervention

After the splints were fabricated, they were placed in the patients’ mouth, and occlusal adjustments were performed. The patients were instructed to wear SS for at least 20 h per day [16]; the device was to be removed during eating and tooth-brushing. Optimal treatment success depended on the patients’ presence in all of their appointments, and several appointments were required to adjust SS. After one week of using SS, a dentist checked the patients’ bite marks on the surface of the splint and made further adjustments by grinding the splint. Doing so helped achieve a stable position of the jaw. Subsequent check-ups were conducted after 15, 30 and 60 days [16]. The patients’ progress was monitored at every appointment, during which the joint area was palpated, muscle tenderness was recorded and, if necessary, the splint was adjusted. The patients were instructed to depend solely on SS for treatment; no medication or physical therapy was applied in conjunction with SS. The evaluation of the treatment after therapy was not based only on patient reports; it also depended on the Helkimo index and Measures Condyle Displacement. These parameters focused on whether the jaw joint was in a stable position, whether the condyles were close to CR and if the symptoms of TMD improved over three follow-up visits [34]. The patients were gradually weaned off the splint by slowly increasing the duration time they went without wearing it. Finally, the patients were instructed to stop wearing the device.

### Three‑dimensional Cbct acquisition

A CBCT unit (KaVo 3D eXam; KaVo Dental, Bismarckring, Germany) was used to acquire 3D images. The CBCT imaging parameters were set to 120 kV and 37.1 mA, with a field of view of 23 × 17 cm, an exposure time of 17.8 s, a voxel size of 0.3 mm and a slice thickness of 0.3 mm. During the procedure, the patients were seated in an upright position with their teeth maximally intercuspated. The scan was performed on the midsagittal plane perpendicular to the floor and the Frankfurt horizontal (FH) plane parallel to it by following a laser guide. All patients were instructed not to swallow during the scan. Data were acquired after the CBCT scan, converted to Digital Imaging and Communication in Medicine file format and imported into Invivo 6 software (Anatomage, San Jose, CA, USA) for 3D analysis.

### Study variables

In this study, the predictor variable is the SS treatment condition, which is categorized into two time points: pre-treatment and post-treatment. The primary outcomes involved the vertical skeletal measurements (LFH, PFH: AFH ratio, MP-SN°, and the sum of the posterior cranial angles) and the vertical dentoalveolar variables (FOP-SN°, FOP-FH°, BOP-SN°, BOP-FH°, and the overbite in mm). The secondary outcome variables were all other variables mentioned in Table 2.

### Sample size calculation

The sample size for the study was determined using G*Power software (version 3.1.9.4) with an alpha value of 0.05 and a power level of 85% on the basis of the study conducted by Derwich et al. [35]. This previous study indicated that the mean change in the mandibular sagittal position after occlusal splint therapy (ANB angle) is 4.4 ± 3.2°. The power analysis revealed that a minimum sample size of 51 patients yields statistical significance. However, the sample size was increased to at least 74 patients.

### Data collection methods

**Fig. 1:**
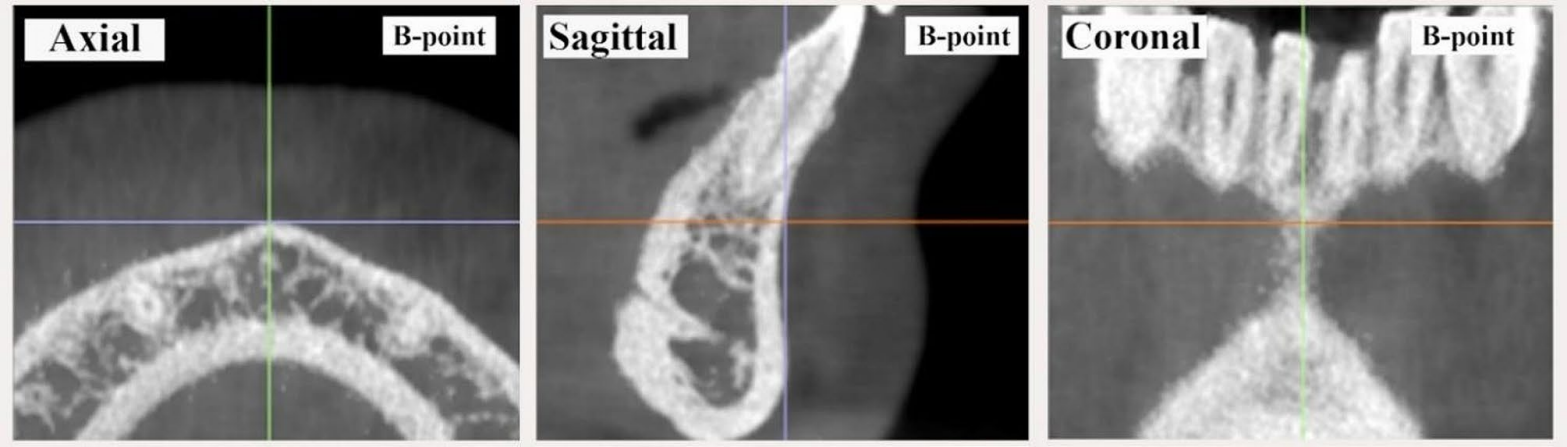
The slice locator determination

**Fig. 2:**
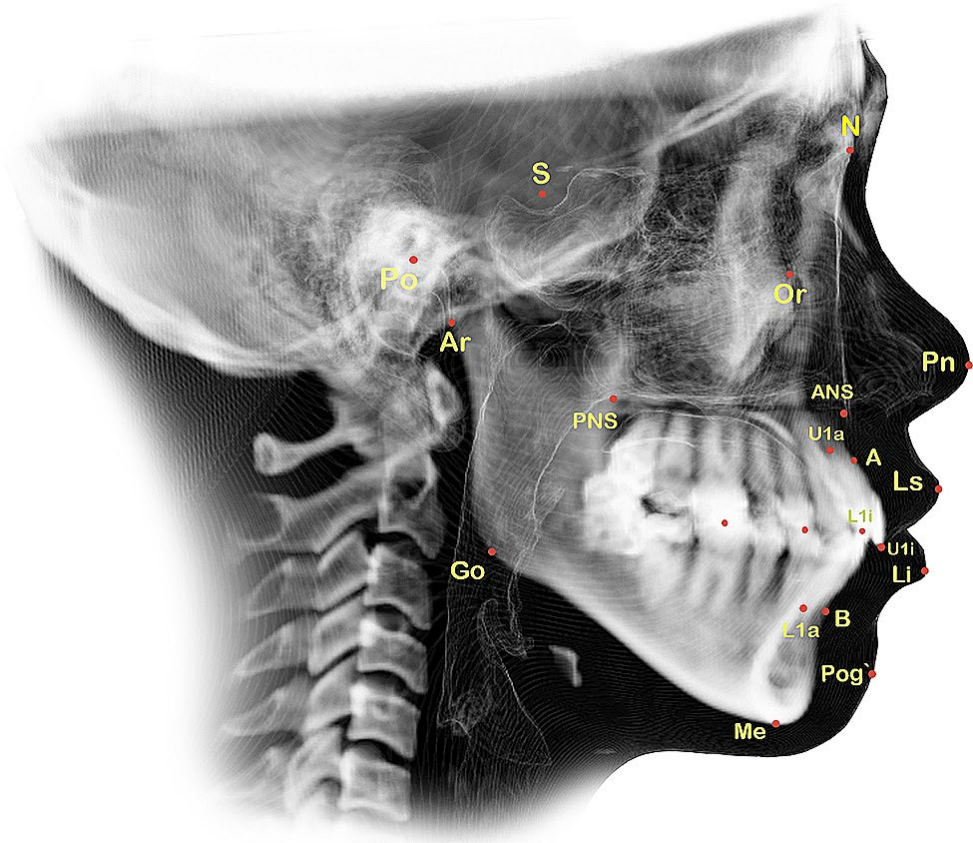
The 3D cephalometric anatomical landmarks

**Fig. 3:**
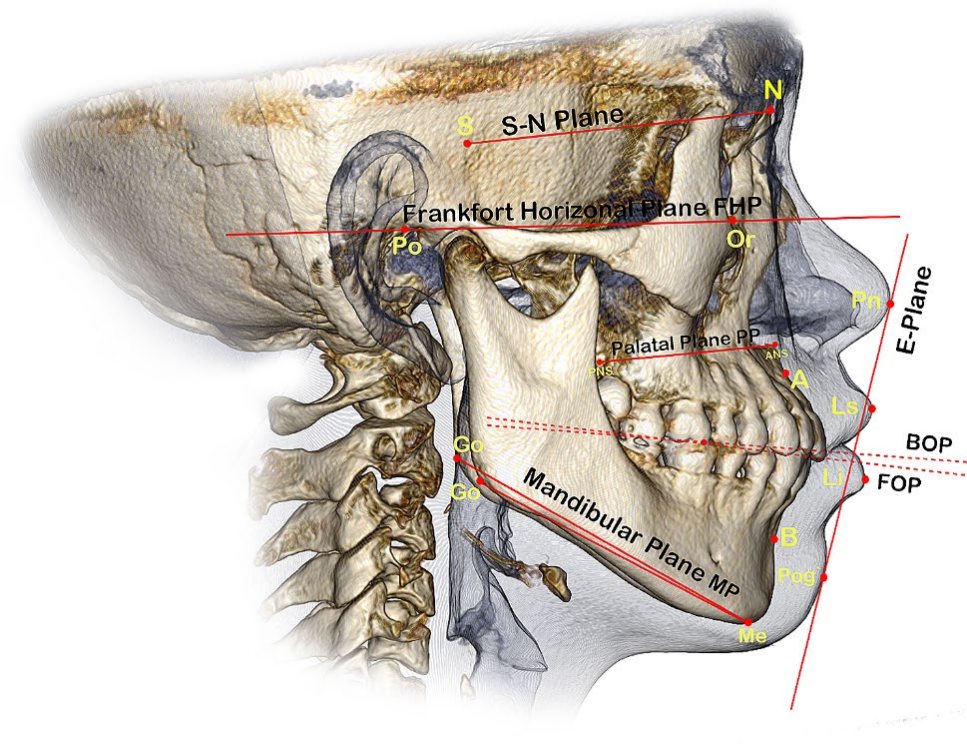
The 3D skeletal reference lines, and planes

The data used in this study were obtained from the complete medical records of patients who were diagnosed and treated from July 2017 to July 2022. The CBCT scans were initially conducted for TMJ diagnosis and subsequently after treatment to assess the treatment’s effectiveness in addressing the identified problem. In this study, the 3D cephalometric method was used to draw and measure points, lines or planes on reconstructed images of CBCT data simulated with Invivo 6.0.3/Anatomage dental software program. Landmarks were digitised based on easily identifiable features that were most prominent in the 3D image. The positions of the selected points were adjusted on the sagittal, coronal and axial planes by using a slice locator, as shown in Fig. 1. Mandibular plane angle (MP-SN) and Jarabak ratio [36] were utilised to investigate the vertical skeletal changes and the effect of SS on mandibular position and angulation after treatment. Bisecting OP (BOP) and functional OP (FOP) were used to measure the angular relationship changes of OP with the Sella–Nasion (SN) and FH planes. According to Downs [37], BOP is a plane that connects the height of the first molar cusp and the point bisecting the incisal overbite; FOP is a plane formed by bisecting the intercuspation of the first molars and premolars [38]. SNA, SNB and ANB were utilised to investigate the anteroposterior skeletal changes after SS treatment. The angulations and positions of the maxillary and mandibular central incisors and the maxillary and mandibular first molars were also measured to evaluate any possible changes and to be consistent with potential changes in OP. In addition, this study assessed anterior overjet and soft tissue measurements, including the distances of the upper and lower lips from the aesthetic plane. The relevant anatomical and constructed landmarks, reference lines and planes and 3D measurements used in this study are shown in Figs. 2 and 3; Tables 1 and 2.

**Table 1:** Definitions of the reference landmarks, lines and planes used in this study

Identification	Abbreviation	Definition
**Anatomical landmark**		
Nasion	N	Anterior- and superior-most point of the frontonasal suture
Sella	S	Sella turcica, which refers to the central area of the hypophyseal fossa located in the middle cranial fossa
Orbital	Or	Right and left inferior-most points on the margin of the orbit
Porion	Po	Right and left highest midpoint on the roof of the external auditory meatus
Articulare	Ar	Right and left points that can be identified where the posterior margin of the ramus intersects with the lower boundary of the cranial base
Gonion	Go	Right and left points of the bisecting angle between the ramus line and the body of the mandible line
Menton	Me	Lowest most inferior point of the symphysis
Subspinale	A	Deepest point of the premaxilla contour
Supramental	B	Deepest point on the anterior mandible contour
Anterior nasal spine	ANS	Most anterior point of the maxilla’s anterior nasal spine
Posterior nasal spine	PNS	Point found at the medial end of the posterior border of the palatine bone horizontal plate
U1 incisal edge	U1i	Upper incisor’s incisal edge
U1 apex	U1a	Upper incisor’s root apex
L1 incisal edge	L1i	Lower incisor’s incisal edge
L1 apex	L1a	Lower incisor’s root apex
Pronasale	Pn	Most protruding nasal tip point
Labrale superior	Ls	Anterior-most point of the upper lip
Labrale inferior	Li	Anterior-most point on the lower lip
Soft tissue pogonion	Pog`	Most protruding point of the chin soft tissue contour
**Reference lines and planes**		
Sella nasion plane	S-N	Line extending from the midpoint of the sella turcica to the nasion point
Palatal plane	PP	Line connecting the anterior and posterior nasal spines
Frankfort horizontal plane	FHP	Horizontal plane that passes through the bilateral orbital and porion points
Mandibular plane	MP	Plane connecting the bilateral gonion and menton
Nasion subspinale line	N-A	Line extending from the nasion to the subspinale point
Anterior facial height	AFH	Line extending from the nasion to the menton point
Posterior facial height	PFH	Line extending from the sella turcica to the midpoint between the two gonions
Bisected occlusal plane	BOP	Plane formed by the lines bisecting the incisal tips, upper and lower anterior teeth and bilateral upper and lower first molar occlusal surfaces
Functional occlusal plane	FOP	Plane constructed by the points bisecting the bilateral occlusal midpoints of the first permanent molars and the midpoints bisecting the intercuspation of the bilateral first premolars
Long axis of the upper or lower incisor	UIx, LIx	Line that extends from the incisor edge to the incisor apex
Aesthetic plane	E-P	Line between the nasal tip’s highest point and the most protruding point of the soft tissue contour of the chin

**Table 2:** Definition of the 3D parameters used in the study

Parameters	Definition
Primary outcome variables (Vertical measurements)	
**Skeletal**	
MP-SN°	Mandibular plane angle created by MP and the S-N plane
LFH (mm)	Lower facial height, which is the distance from the ANS anterior nasal spine to the lowest point on the chin menton point
PFH: AFH (%)	Facial height ratio, which is the proportion between posterior facial height and anterior facial height
Saddle angle°	Angle formed by landmarks N, S and Ar
Articular angle°	Angle formed by landmarks S, Ar and Go
Gonial angle°	Angle formed by landmarks Ar, Go and Me
Upper gonial angle°	Angle created by landmarks Ar, Go and N
Lower gonial angle°	Angle created by landmarks N, Go and Me
SUM:	Sum of the three posterior angles, namely, saddle, gonial and articular angles
**Dentoalveolar**	
FOP-SN°	Angle created by the S-N plane and FOP
BOP-SN°	Angle created by the S-N plane and BOP
FOP-FH°	Angle created by the FH plane and FOP
BOP-FH°	Angle created by the FH plane and BOP
Overbite (mm)	Distance measured vertically from the incisal edges of the upper central incisor to the incisal edges of the lower central incisor
**Secondary outcome variables (Anteroposterior measurements)**	
**Skeletal**	
SNA°	Angle created by landmarks S, N and A
SNB°	Angle created by landmarks S, N and B
ANB°	Angle created by landmarks A, N and B
**Dentoalveolar**	
Overjet (mm)	Distance measured horizontally from the incisal edges of the upper central incisor to the incisal edges of the lower central incisor
U1/L1°	Inter-incisal angle created by the upper and lower incisors’ long axis
U1-SN°	Angle created by the upper incisor’s long axis and the S-N plane
U1-NA°	Angle created by the upper incisor’s long axis and the N-A line
U1-NA (mm)	Distance from the incisal tip of the upper incisors to the N-A line
U1-PP mm	Distance measured perpendicularly from the upper incisor’s tip to the palatal plane
U6-PP mm	Distance measured perpendicularly from the tip of the mesiobuccal cusp of the first upper molar to the palatal plane
L1-NB°	Angle created between the lower incisor’s long axis and the N-B line
L1-NB (mm)	Distance measured between the incisal tip of the lower incisors and the N-B line
L1-MP°	Angle created between the long axis of the lower incisor and the mandibular plane
L1-MP mm	Perpendicular distance from the tip of the lower incisor to the mandibular plane
L6-MP mm	Distance measured perpendicularly from the mesiobuccal cusp’s tip in the lower first molar to the mandibular plane
**Soft Tissue**	
UL-EP (mm)	Distance from the labrale superior (Ls) point to the aesthetic plane
LL-EP (mm)	Distance from the labrale inferior (Li) point to the aesthetic plane

3D (three-dimensional), mm (millimeters), ° (degree), % (ratio measurements)

### Data analyses

Statistical analysis of the data was performed using the Statistical Package for Social Sciences (SPSS) version 25.0 software for Windows (IBM Corp., Armonk, NY, USA). The normality of the data was assessed using the Shapiro–Wilk test. Descriptive statistics of the mean and standard deviation (SD) were provided for each of the 74 patients, and the significance level of the results was set to *p* < 0.05. Paired t-test and Wilcoxon’s rank-sum test were used to determine the statistical significance of the mean changes between pre- and post-treatment measurements. The intraclass correlation coefficient and the absolute and relative technical errors of measurement tests (TEM and rTEM) were used to determine a measurement’s reproducibility and reliability.

## Results

In this study, we included 74 adult patients (25 males and 49 females), with an average age of 22.88 ± 4.8 years. The patients underwent treatment for a period of 6 to 12 months, with an average treatment duration of 9.8 months.

The descriptive statistics, differences and significant (p) values for all primary and secondary outcome variables used in the study are shown in Tables 3 and 4, respectively. Excellent intra- and interobserver reliability was found, the intra-observer relatability ranged from 0.962 to 0.999 for the 31 measurements and the interobserver reliability ranged from 0.952 to 0.999 for the 31 measurements.

**Table 3:** Pre-and post-treatment descriptive statistics, differences and significant p-values for the primary outcome variables (Vertical measurements) used in the study

Measurements		Pre-treatment M ± SD	Post-treatment M ± SD	Differences M ± SD	*P* value	
**Skeletal**						
MP-SN°		34.73 ± 5.96	35.54 ± 6.01	0.82 ± 1.37	0.000***	
LFH (mm)		65.52 ± 5.21	66.14 ± 5.56	0.62 ± 1.89	0.006**	
PFH: AFH (%)		66.76 ± 4.47	66.31 ± 4.57	-0.45 ± 1.07	0.001**	
N-S-Ar°		121.39 ± 5.76	121.05 ± 5.75	-0.34 ± 1.55	0.061	
S-Ar-Go°		150.54 ± 7.12	151.70 ± 6.61	1.16 ± 3.68	0.008**	
Ar-Go-Me°		122.85 ± 6.50	122.99 ± 6.72	0.14 ± 1.62	0.469	
Ar-Go-N°		45.02 ± 3.64	44.72 ± 3.59	-0.30 ± 1.41	0.069	
N-Go-Me °		77.83 ± 5.03	78.27 ± 5.26	0.44 ± 1.06	0.001**	
SUM°		394.78 ± 6.15	395.73 ± 6.37	0.95 ± 2.57	0.002**	
**Dentoalveolar**						
FOP-SN°		19.48 ± 5.14	19.86 ± 4.71	0.38 ± 1.43	0.026*	
BOP-SN°		16.92 ± 4.83	17.36 ± 4.52	0.44 ± 1.29	0.005**	
FOP-FH°		10.02 ± 5.35	10.51 ± 4.91	0.49 ± 1.62	0.012*	
BOP-FH°		7.67 ± 4.42	8.08 ± 4.08	0.41 ± 1.17	0.003**	
Overbite (mm)		3.79 ± 1.64	3.25 ± 1.60	-0.54 ± 0.83	0.000***	

M, mean value; SD, standard deviation; mm (millimeters); °(degree); % (ratio measurements)

*P < 0.05; **P < 0.01; ***P < 0.001

**Table 4:** Pre-and post-treatment descriptive statistics, differences and significant p-values for the secondary outcome variables (Anteroposterior measurements) used in the study

**Measurements**	**Pre-treatment** *M ± SD*		**Post-treatment** *M ± SD*	**Differences** *M ± SD*		***P*** **value**
**Skeletal**						
SNA°	82.53 ± 3.93		82.82 ± 3.82	0.29 ± 1.37		0.075
SNB °	79.62 ± 3.45		78.02 ± 3.27	-1.60 ± 1.36		0.000***
ANB°	2.91 ± 2.88		4.80 ± 2.82	1.89 ± 1.61		0.000***
**Dentoalveolar**						
Overjet (mm)	4.07 ± 2.57		5.00 ± 2.79	0.93 ± 1.04		0.000***
U1/L1°	124.59 ± 13.02		123.57 ± 12.79	-1.02 ± 2.42		0.001**
U1-SN°	74.47 ± 9.15		74.30 ± 9.16	-0.18 ± 2.63		0.566
U1-NA°	23.44 ± 7.85		23.54 ± 7.72	0.09 ± 2.56		0.754
U1-NA (mm)	4.92 ± 2.25		5.04 ± 2.28	0.13 ± 0.88		0.218
U1-PP (mm)	28.33 ± 2.82		28.21 ± 2.76	-0.12 ± 0.74		0.153
U6-PP (mm)	23.48 ± 2.53		23.39 ± 2.47	-0.09 ± 0.63		0.222
L1-NB°	28.94 ± 8.06		29.39 ± 7.98	0.44 ± 1.37		0.007**
L1-NB (mm)	6.42 ± 3.03		6.60 ± 2.93	0.18 ± 0.92		0.092
L1-MP°	94.62 ± 9.11		94.92 ± 9.12	0.30 ± 1.81		0.160
L1-MP mm	40.20 ± 3.45		40.15 ± 3.81	-0.06 ± 1.92		0.804
L6-MP mm	31.52 ± 2.74		31.64 ± 2.96	0.12 ± 1.32		0.439
**Soft tissue**						
UL-EP (mm)	2.77 ± 2.13		2.79 ± 2.18	0.02 ± 2.40		0.954
LL-EP (mm)	3.42 ± 1.65		3.75 ± 1.99	0.33 ± 1.01		0.006**

M, mean value; SD, standard deviation; mm (millimeters); °(degree); *P < 0.05; **P < 0.01; ***P < 0.001

### Primary outcome

#### Vertical skeletal measurements

According to the analysis of the pre- and post-treatment 3D cephalometric measurements, a highly significant difference (*p* < 0.001) was observed in the angle between the mandibular and SN planes, which increased by a mean of 0.82 ± 1.37°. The Jarabak ratio (PFH: AFH) exhibited a statistically significant decrease (*p* < 0.01), with an average of 0.45 ± 1.07%. This finding is consistent with the significant increase noted in the lower facial height (*p* < 0.01; average: 0.62 ± 1.89 mm). Furthermore, the articular angle exhibited a statistically significant increase (1.16 ± 3.68°, *p* < 0.01), but no significant difference was observed in the saddle angle. The upper gonial angle decreased but not significantly, and the lower gonial angle increased significantly (0.44 ± 1.06°, *p* < 0.01).

### Vertical dentoalveolar measurements

The occlusal planes measurements presented a statistically significant increase in the inclinations of FOP-SN, FOP-FH, BOP-SN and BOP-FH; the average differences were 0.38 ± 1.43°, 0.49 ± 1.62°, 0.44 ± 1.29° and 0.41 ± 1.17°, respectively (*p* < 0.01 for FOP and *p* < 0.05 for BOP). A significant decrease (*p* < 0.001) in overbite was observed, with an average of 0.54 ± 0.83 mm.

### Secondary outcome

#### Anteroposterior skeletal measurements

The SNA angle had no statistically significant variations. However, the SNB angle exhibited a significant reduction (*p* < 0.001), and the mean difference was 1.60 ± 1.36°. The ANB angle showed a significant increase (1.89 ± 1.61°, *p* < 0.001).

### Anteroposterior dentoalveolar measurements

A significant increase in overjet was observed (*p* < 0.001), with an average of 0.93 ± 1.04 mm. Furthermore, a significant decrease in the U1/L1 angle was found (1.02 ± 2.42°; *p* < 0.01). The study’s results indicate that there were no significant changes in the inclination of U1 (U1-SN, U1-NA), the U1-NA distance, L1-MP inclination, or L1-NB distance. However, statistically significant changes were observed in the inclination of L1-NB; the mean difference was 0.44 ± 1.37° (*p* < 0.01). Additionally, the positions of U1, U6, L1 and L6 relative to PP and MP did not show any statistically significant differences.

### Soft tissue measurements

There are no significant changes were observed in the upper lip’s distance from the aesthetic line. However, the lower lip’s distance exhibited significant changes (*p* < 0.01) and was characterised by a mean of 0.33 ± 1.01 mm.

## Discussion

TMD is one of the most common oral and maxillofacial disorders [39]. It can be treated with occlusal splints, manual therapy, physiotherapy, counselling therapy, arthrocentesis or arthroscopy, and oral or injectable pharmacotherapy. Occlusal splints are commonly used as a non-invasive treatment for patients with TMD. Among occlusal splint types, SS is one of the most effective [13], and has been shown to have an acceptable treatment effect on the signs and symptoms of TMD patients [11]. In this retrospective study, three-dimensional cephalometric analyses were performed using CBCT and primarily examined the vertical skeletal and dentoalveolar changes and secondarily the anteroposterior skeletal, dentoalveolar, and soft tissue changes after SS therapy in patients with myofascial and/or intra-articular disorders.

In terms of the primary outcome variables, this study demonstrated an increased steepness of the mandibular plan as represented by the increase in the MP-SN angle after SS treatment. Previous case study reports have shown that occlusal changes and clockwise mandibular rotation with facial profile changes often occur after splint therapy for patients with TMDs; these changes can be attributed to alterations in condylar position and morphology, and they can further worsen the condition of a retrognathic mandible and complicate subsequent orthodontic treatments [29, 31].

The comparison of the results of the Jarabak ratio before and after treatment revealed a significant decrease in the ratio of posterior facial height to anterior facial height, which is consistent with the increase in the lower facial height and the mandibular angle. In addition, the articular angle increased, and the lower gonial angle increased, indicating that the mandible shifted posteriorly in relation to the skull and that the mandible underwent clockwise rotation. These changes may explain the increased overjet, decreased overbite and open occlusion that were observed after treatment. The presence of these occlusal changes after SS treatment might be due to the restoration of the mandible to the CR position. Furthermore, the inability to achieve the same occlusion as that before SS treatment revealed that articular remodelling and adaptive changes may have occurred, as described in literature [40, 41].

The inclination of OP can be altered by various factors, such as ageing, growth, occlusal wear, tooth loss and use of dental devices [42, 43]. Changes in OP may also depend on changes in the reference planes, such as SN and FH. The findings of this study reveal a significant increase in the inclination of the functional and bisecting OPs, and these observed changes may be attributed to the clockwise rotation of the mandible after SS treatment. Additionally, the initial occlusal contact in the molar region may change with the disocclusion of the molars, which often occurs due to the posterior displacement of the mandible when the CR of the patient is taken and the condyles are seated in the CR position, which may affect the inclination of OP. In the study of Magdaleno and Ginestal [29], a patient experienced unstable occlusions with dental contacts only at the level of the wisdom teeth upon removal of a splint. Subsequently, the patient was informed of the need for orthodontic treatment to stabilise the occlusions, and the patient was referred to an orthodontist.

The current study findings revealed a significant decrease in the overbite. Alterations in the occlusal relationship induced by wearing SS may lead to the development of an anterior open bite. Intrusion of the molars is considered one of the most effective treatment methods for correcting an open bite [44]. In severe cases where patients already have an open bite before treatment and it increases further after SS treatment, orthognathic surgery may be recommended to correct such skeletal discrepancies after splint treatment [45]. When a splint is used, the condyle in the CR sits passively in the fossa, which can result in altered dental occlusion, with contact primarily on the most posterior teeth and the potential development of an anterior open bite [25, 29].

Changes in jaw position have a considerable impact on the diagnosis and treatment of dental malocclusion. These changes directly affect the diagnosis of a patient’s vertical skeletal pattern and the dentist’s analysis of the treatment mechanism. This impact can be more complex and challenging in patients with a vertical skeletal pattern than in patients with a horizontal skeletal pattern, as indicated by Shildkraut et al. [46]. The current study showed that the skeletal patterns of patients undergoing SS treatment tended to change from the original horizontal type to the average type or from the average type to the vertical type. This finding is important for determining whether an orthodontic treatment plan should include tooth extraction or orthognathic surgery to correct skeletal discrepancies, which is especially true in cases that require orthodontic treatment after SS treatment, as reported in some studies [31, 45]. In summary, the position of the mandible plays a crucial role in determining the appropriate clinical treatment plan.

Patients with a steep maxillary OP are likely to experience occlusal interference during mandibular movement and develop a convex facial profile. The steeper the maxillary OP is, the greater the impact force is during tooth contact. Early contact points on mandibular teeth may act as a fulcrum, potentially leading to condyle displacement [31, 45].

Regarding the secondary outcome variables, the current study findings revealeda significant retrusion of the mandible (decrease in SNB angle), a significant increase in the sagittal discrepancy between both jaws (↑ANB angle) and a significant increase in the overjet. No significant maxillary or mandibular dentoalveolar changes were found in this study. This result reveals that SS might not have an effect on the angulation and position of the incisors and molars. The exception is the angulation of L1-NB, which increased significantly due to the posterior displacement and clockwise rotation of the mandible. Moreover, the current study’s results showed a significant increase in LL-EP distance, which is consistent with the results of increased ANB angle due to changes in mandibular position. This change in LL-EP distance may have affected the patients’ profiles after SS treatment.

Patients undergoing single-stage treatment with occlusal splints may experience a relapse of symptoms after treatment [47]. Thus, dentists sometimes recommend that the treatment be performed in two stages. In the first stage, an occlusal splint is used, followed immediately by the second stage of orthodontic treatment to correct the position of the teeth, correct the rotation of the mandible and eliminate all occlusal disturbances. The goal is to obtain an improved long-term curative outcome, as reported by Ramachandran et al. [48]. A stable jaw after splint therapy ensures stable orthodontic treatment results [49]. However, this recommendation is not generalisable to all patients undergoing SS therapy. It applies only to patients who experience severe post-treatment effects. Therefore, post-SS treatment analyses must be performed to determine the appropriate course of action.

The study’s findings suggest crucial considerations for the management of TMD using stabilization splints. The observed possible post-treatment changes in mandibular position and occlusion emphasize the necessity for careful monitoring and individualized treatment planning. Clinicians must remain attentive to potential changes such as skeletal discrepancies and anterior open bite, which may necessitate further orthodontic interventions to achieve optimal and stable post-TMD treatment results. These changes not only have immediate implications for treatment but also carry significance for diagnosis and subsequent treatment planning if required.

Limitations of this study include its retrospective design, which prevents blinding and the inclusion of a control group. The need for a larger sample size to enhance the generalizability and validity of the findings with more males to be included to analyze the effect of gender, and the pre-treatment skeletal vertical patterns on the treatment changes. Additionally, conducting long-term follow-up studies to assess treatment effects after considerable period of time.

## Conclusion

After SS treatment, patients may undergo considerable changes, such as downward and backward movements of the mandible, increase in OP inclination, possibility of developing an anterior open bite and profile variations. These changes may indicate pre-existing positional discrepancies in patients’ joints. Therefore, these possible posttreatment changes must be fully considered to maintain the stability of the results and enable improved diagnoses and orthodontic treatment planning, if required.

## Data Availability

The data supporting the findings of this study are available from the corresponding author upon reasonable request.

## Abbreviations

TMD: Temporomandibular joint disorders
TMJ: Temporomandibular joint
SS: Stabilization splints
3D: Three dimensions
CBCT: Cone‑beam Computed Tomography
FOP: Functional occlusal plane
BOP: Bisecting occlusal plane
